# Supplementary material for: Comprehensive Landscape of BRAF Variant Classes, Clonalities, and Co-Mutations in Metastatic Colorectal Cancer Using ctDNA Profiling
Source: Cancers (Basel). 2024 Feb 9;16(4):737. doi: 10.3390/cancers16040737 (PMC10886949; doi:10.3390/cancers16040737)
Supplement: Supplementary file 1 [file cancers-16-00737-s001.zip › cancers-2774136-supplementary.pdf]

Table S1: Summary of the clinical cohort patients' characteristics

		N (%)
Age, median (range)		55 (31, 94)
Ethnicity	Caucasian	30 (81.1%)
	Hispanic	5 (13.5%)
	African American	1 (2.7%)
	Asian	1 (2.7%)
Sex	F	19 (50%)
	M	19 (50%)
Primary Location	ascending	4 (10.5%)
	cecum	5 (13.2%)
	rectal	17 (44.8%)
	sigmoid	9 (23.7%)
	transverse	3 (7.9%)
Side	left	26 (74.3%)
	right	9 (25.7%)
Lines of Treatment	1	6 (15.8%)
	2	14 (36.8%)
	3	7 (18.4%)
	4	10 (26.3%)
	5	1 (2.6%)
Peritoneum involved	No	27 (73%)
	Yes	10 (27%)
Liver involved	No	11 (29.7%)
	Yes	26 (70.3%)
Lung involved	No	15 (40.5%)
	Yes	22 (59.5%)
Lymph node	No	17 (45.9%)
	Yes	20 (54.1%)
Other site	No	30 (81.1%)
	yes; bone, ovary	1 (2.7%)
	yes; ovary	2 (5.4%)
	yes; bone	4 (10.8%)
Metastasectomy	No	28 (73.7%)
	Yes	10 (26.3%)
Site of Metastasectomy	liver	5 (50%)
	lung	3 (30%)
	ovary	1 (10%)
	peritoneal/pelvic nodule	1 (10%)
Anti-EGFR treatment	No	25 (65.8%)
	Yes	13 (34.2%)
